# Comparison of Measured and Estimated Resting Energy Expenditure in Adolescents and Young Adults With Severe Obesity Before and 1 Year After Sleeve Gastrectomy

**DOI:** 10.3389/fped.2019.00037

**Published:** 2019-02-19

**Authors:** Frances A. Rickard, Landy P. Torre Flores, Sonali Malhotra, Alexander T. Toth, Abeer Bader, Fatima Cody Stanford, Hang Lee, Miriam A. Bredella, Madhusmita Misra, Vibha Singhal

**Affiliations:** ^1^Melbourne School of Medicine, University of Melbourne, Melbourne, VIC, Australia; ^2^Neuroendocrine Unit, Massachusetts General Hospital and Harvard Medical School, Boston, MA, United States; ^3^Pediatric Endocrinology, Massachusetts General Hospital and Harvard Medical School, Boston, MA, United States; ^4^MGH Weight Center and Harvard Medical School, Boston, MA, United States; ^5^Biostatistics Unit, Massachusetts General Hospital and Harvard Medical School, Boston, MA, United States; ^6^Department of Radiology, Massachusetts General Hospital and Harvard Medical School, Boston, MA, United States

**Keywords:** obesity, adolescents, resting energy expenditure, calorimetry, equation, sleeve gastrectomy

## Abstract

**Introduction:** Resting energy expenditure (REE) is often evaluated in adults and adolescents with obesity to estimate caloric requirements when advising dietary changes. However, data are lacking regarding the accuracy of methods used to clinically assess REE in adolescents with severe obesity. Moreover, there are no data regarding the effects of sleeve gastrectomy (SG) on REE in adolescents. We evaluated the accuracy and error rate between estimated and measured REE in adolescents with severe obesity and changes in REE following (SG).

**Materials and Methods:** *Cross-sectional study* (CSS): 64 adolescents and young adults, 14–22 years old, with moderate to severe obesity were enrolled. We measured REE (mREE) by indirect calorimetry and estimated REE (eREE) using Derumeaux (Deru), Mifflin-St Jeor (MS), Harris Benedict (HB), and World Health Organization (WHO) equations. DXA was used to determine body composition. Bland Altman analysis evaluated agreement between eREE and mREE. *Longitudinal study*: 12 subjects had repeat indirect calorimetry and DXA 1 year after SG. Longitudinal analysis was used to assess changes in REE and body composition.

**Results:** *CSS*: Median BMI was 45.2 kg/m^2^ and median age was 18.0 (16.3–19.9) years. mREE correlated strongly with eREE . Bland Altman analysis demonstrated that only a few points were beyond the 1.96 SD limit of disagreement. However, there was considerable overestimation of mREE by most equations. *Longitudinal Study*: In the subset that underwent SG, after 12-months, absolute REE decreased from 1709 (1567.7–2234) to 1580.5 (1326–1862.5) Calories (*p* = 0.002); however, the ratio of REE/Total Body Weight (TBW) increased from 13.5 ± 2.3 at baseline to 15.5 ± 2.2 at 1 year (*p* = 0.043). When evaluating parameters affecting % total weight loss, we found that it correlated positively with REE/TBW at 12 months (*R* = 0.625; *p* = 0.03) and negatively with % fat mass at 12 months (*R* = −0.669; *p* = 0.024).

**Discussion:** In adolescents with moderate-severe obesity, despite a correlation between mREE using indirect calorimetry and eREE using the Deru, MS, HB, and WHO equations, there is significant over-estimation of REE at the individual level, challenging their clinical utility. One year after SG, REE/TBW increased and strongly correlated with % total weight loss in adolescents.

## Introduction

Childhood and adolescent obesity is a growing public health concern. Currently, more than 20% of children in the United States have obesity, placing them at increased risk of obesity and its comorbidities as adults (1).

While obesity is a multifactorial disease, it is ultimately the result of a state of positive energy balance combined with a genetic predisposition for weight gain (2). To address energy imbalance, current recommendations for treating childhood obesity include family based lifestyle interventions with diet and exercise modifications (3). As part of this current strategy, energy requirements are often measured to guide the development of appropriate recommendations to achieve weight loss or maintenance. Indirect calorimetry is the current gold standard to measure resting energy expenditure (REE), but this technique is expensive and often difficult to access in clinical practice. Equations that estimate REE have been developed, however their accuracy in children with obesity is questionable, which makes it difficult to rely on these estimates in a clinical setting for this patient population (4).

Bariatric surgery is a useful treatment option for adolescents with severe obesity (5). When weight loss is achieved through diet and exercise, there is an associated decrease in REE (6, 7), which makes continued weight loss challenging. In adults, bariatric surgery results in decreases in absolute REE, but this decrease in REE is not evident when controlled for total body weight, suggesting that REE actually does not change (7). There are only a few studies in adults that have evaluated changes in REE after sleeve gastrectomy (SG), and results are conflicting (8, 9). To our knowledge, there are no studies that have assessed metabolic changes in adolescents after SG.

The primary objectives of this study are to (i) evaluate the accuracy of predictive equations used in clinics to estimate REE in adolescents with moderate to severe obesity, and (ii) to explore metabolic adaptations in adolescents and young adults with severe obesity 12 months following SG. We hypothesized that REE predictive equations would be inaccurate in adolescents with severe obesity, and that SG would lead to metabolic adaptations, causing a reduction in REE.

## Methods

This study was carried out in accordance with the Belmont principles and was approved by the Institutional Review Board of Partners Healthcare System. All subjects gave written informed consent in accordance with the Declaration of Helsinki. Subjects ≥18 years of age, and parents of subjects <18 years of age provided written informed consent, and informed assent was obtained from subjects <18 years of age. Sixty-four adolescents and young adults, ranging from 14–22 years of age, with moderate to severe obesity, BMI > 35 kg/m^2^ were enrolled. Exclusion criteria included untreated thyroid dysfunction, smoking >10 cigarettes/day, substance abuse disorder, pregnancy or breast feeding as these effect REE. Four of our participants were on medications for attention deficit hyperactivity disorder (ADHD)—three were on dextroamphetamine and one on methylphenidate. Although, considered stimulant drugs, literature suggests that these medications do not alter REE (10). We did not exclude adolescents on stimulant medications given the frequency of their use in the general population, as this would limit the generalizability of our results. No participant was on stimulant medications for weight loss.

## Measurements

Indirect calorimetry (MetCart) was used to measure fasting REE under thermal neutrality using VMAX Encore 29 metabolic cart (Viasys Healthcare, Carefusion; San Diego, CA). All measurements were conducted at the Clinical Research Center of our institution. Subjects fasted for at least 8 h before measurement and were instructed to refrain from heavy exercise, tobacco, and alcohol the night before. Subjects did not alter their usual food intake the day prior to the visit. They rested for 20 min before measurement. Dual energy x-ray absorptiometry (Hologic QDR 4500) was used to measure body composition, specifically total fat and lean mass. Activity levels were recorded using the Paffenberger questionnaire.

Four REE equations were used to compare measured vs. estimated REE. These included the Derumeaux, Harris Benedict, World Health Organization/Food and Agriculture Organization and Mifflin- St Jeor equations (Table 1). These equations were chosen because clinically they are the most commonly used equations, and/or because younger populations were included in their initial derivation. Twelve subjects underwent SG and had repeat indirect calorimetry and DXA measurements 12 months after surgery.

**Table 1:** REE equations evaluated in adolescents with moderate-severe obesity.

**Author**	**Sex**	**Age**	**Equations to estimate resting energy expenditure**
Derumeaux-Burel et al. (11)	Male		*REE* = 0.1096 × *FFM* + 2.8862
	Female		*REE* = 0.1371 × *FFM* − 0.1644 × *age* + 3.3647
Harris et al. (12)	Male		*REE* = 66.473 + (13.752 × *W*) + (5.003 × *H*) − (6.755 × *age*)
	Female		*REE* = 665.096 + (9.5634 × *W*) + (1.849 × *H*) − (4.6756 × *age*)
Mifflin et al. (13)	Male		*REE* = (9.99 × *W*) + (6.25 × *H*) − (4.92 × *age*) + 5
	Female		*REE* = (9.99 × *W*) + (6.25 × *H*) − (4.92 × *age*) − 161
WHO (14)	Male	>18	*R EE* = 15.3 × *W* + 679
		< 18	*EE* = 17.5 × *W* + 651
	Female	>18	*REE* = 15.3 × *W* + 496
		< 18	*REE* = 12.2 × *W* + 746

*REE, resting energy expenditure; Deru, Derumeaux; WHO, World Health Origination; FFM, free fat mass; kg, W, weight, kg; H, height; cm, age in years*.

## Statistical Analysis

JMP Pro12 (SAS Institute, Cary, NC, USA) was used for data analysis. The variables were assessed for normality of distribution and descriptive analysis is presented accordingly. The percentage of subjects with accurate prediction of REE on an individual level was also calculated, where an accurate prediction was defined as the estimated REE being between 90 and 110% of the measured REE (based on previous studies and clinical impact) (15). If the eREE was < 90% of mREE, this was defined as an under prediction, whereas if the eREE was >110% of the mREE this was considered an over prediction. The percentage of the maximum positive/maximum negative error refers to the maximum positive and negative individual value of eREE that was the furthest away from the mREE (over and under prediction, respectively). Correlation is simple linear correlation between eREE and mREE. Bland Altman plots were used to evaluate the agreement between four predictive equations and measured REE (16).

For the longitudinal component of the study, we used the paired samples *t*-test or the Wilcoxon signed rank test, depending on distribution, to test if there was a significant within- group change over 12 months. For correlational analysis, Pearson's or Spearman's correlation was used depending on data distribution.

## Results

### Subject Characteristics

The demographic and anthropometric characteristics of the 64 adolescents and young adults with moderate to severe obesity included in this study are displayed in Table 2. Almost half of our participants were Caucasians and 40% were Hispanic in ethnicity. The median hours of vigorous activity per week was 3.8 (0–7).

**Table 2:** Subject characteristics.

	***N* = 64**
Age, years	18.0 (16.3–19.9)
Sex	16 Male, 48 Female
**RACE**	
Caucasian (%)	48.4
African American (%)	21.9
More than one race (%)	12.5
American Indian (%)	3.1
Unknown (%)	12.5
**ETHNICITY**	
Hispanic (%)	40.6
Non-Hispanic (%)	59.4
Weight, kg	125.3 (111.9–144.9)
Height, cm	167.5 ± 7.7
BMI, kg/m^2^	45.2 (40.4–49.0)
% Ideal BMI for age	209.6 (190.1–229.8)
% Fat Mass	49.8 (45.7–52.3)
% Lean Mass	48.8 (46.8–53.1)
Vigorous exercise, h/wk	3.8 (0.0–7.0)
Sleeping hours, h/wk	54.2 ± 11.8

*Values are presented as median (IQR) or mean ± SD or as a % of total subjects. BMI, body mass index*.

### Accuracy of REE Predictive Equations

Table 3 shows the difference between measured REE using indirect calorimetry and predicted REE using the four representative predictive equations (Table 1). The percentage of accuracy, over prediction, bias, maximum positive and negative error are listed. The Mifflin equation had the lowest difference from the mREE with a mean difference of 303.6 kcal/day. The WHO equation was the furthest from the mREE, showing a mean difference 683.3 kcal/day. Over all, the Mifflin equation performed best in our subjects with the lowest bias (18.9%), and an accurate prediction of REE in 25% of subjects. The WHO equation performed the poorest showing the highest bias (39.3%) and giving an accurate prediction in only 3.1% of subjects. The maximum positive error was estimated using the WHO equation, where one subject was predicted to have an REE that was 87.4% higher than the measured value. More than a third of the time, all four equations over predicted REE.

**Table 3:** Comparison of measured REE and estimated REE in adolescents with moderate-severe obesity.

**Predictive equations**	**Absolute difference (eREE -mREE) (Kcal/day)**	**Accurate predictio*n* (%)**	**Under prediction (%)**	**Over prediction (%)**	**Bias (%)**	**Maximum negative error (%)**	**Maximum positive error (%)**	Correlation with mREE^*^
Deru	480.7	14.1	1.6	84.4	27.9	−13.5	62.2	0.70
HB	435.3	9.4	1.6	89.1	25.6	−15.5	70.8	0.78
Mifflin	303.6	25	1.6	73.4	18.9	−15.7	48.8	0.80
WHO	683.3	3.1	0	96.9	39.3	NA	87.4	0.78

*Accurate prediction: percentage of patients predicted by equation within 10% of measured value*.

*Under prediction: percentage of patients predicted by this equation < 10% of measured value*.

*Over prediction: percentage of patients predicted by this equation> 10% of this measure value*.

*Bias: mean percentage error between predictive equation and measured value*.

*EREE, estimated resting energy expenditure; MREE, measured resting energy expenditure; Deru, Derumeaux; HB, Harris Benedict; WHO, World Health Organization*.

* *P**-**value for all correlation < 0.0001*.

**Figure 1:**
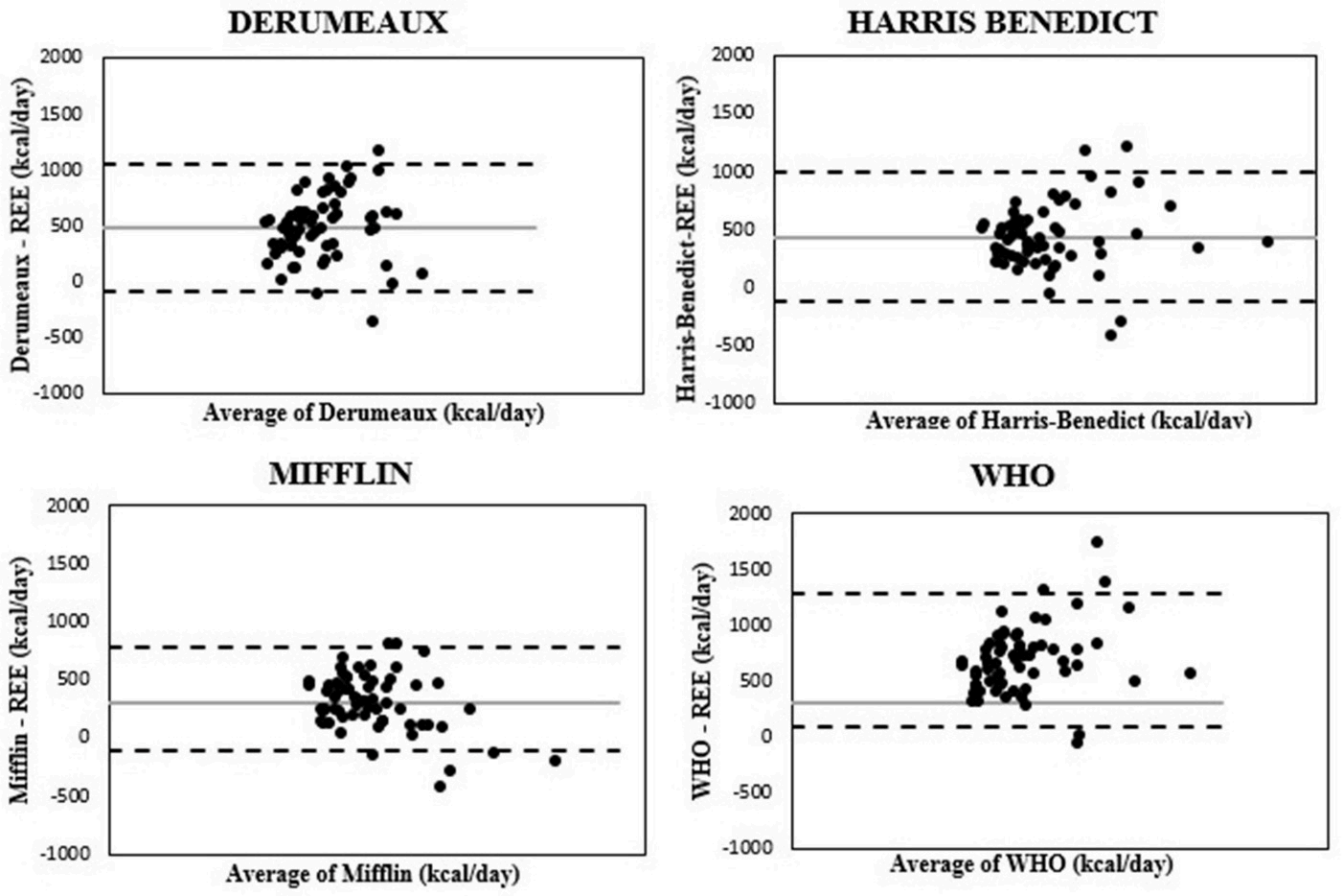
Bland-Altman plots comparing absolute differences between measured and estimated REE and average of measured and estimated REE for the four equations. 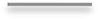, Mean; 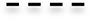, +/−1.96SD, 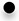
Subject.

Bland Altman plots, Figure 1, shows the distributions of differences against the means, obtained with the two different methods, and the limits of agreement. Most subjects were within the suggested limits of agreement across the four equations, and there was no evidence of dependency of the differences in the entire range of energy expenditure levels suggesting the lack of systematic error.

### REE and Metabolic Changes After Sleeve Gastrectomy

Weight, body composition and metabolism changes from baseline to 12 months in adolescents and young adults undergoing SG (*N* = 12) are shown in Table 4. The group was composed of mostly white (9/12) females. About 42% were of Hispanic ethnicity. The mean age baseline was 18.8 ± 2.2. As expected, they had a decrease in weight, BMI, total and % fat mass and total lean mass. Although the group showed a decrease in absolute REE, REE was unchanged after controlling for lean mass. Further, REE/total body weight increased 12 months after SG.

**Table 4:** Anthropometric and metabolic changes 1 year after sleeve gastrectomy.

**Characteristics**	**Pre-surgical**	**Post-surgical**	***P*-value**
Weight (kg)	143.5 ± 30.4	100.1 (90–115.5)	**0.0005**
Excess body weight (%)	53.2 ± 6.9	41.4 ± 13.7	**0.0005**
BMI (kg/m2)	50.1 ± 8.6	33.9 (31–53)	**0.034**
Fat mass (kg)	69.7 ± 13.1	44.9 ± 11.5	**0.001**
% Fat mass	50.4 ± 3.8	43.7 ± 6.1	**0.005**
Lean Mass (kg)	65.7 ± 9.6	55.3 ± 8.6	**0.001**
% Lean mass	47.8 ± 3.6	54.6 ± 5.6	**0.003**
REE (kcal/day)	1709 (1567.7–2234)	1580.5 (1326–1862.5)	**0.002**
REE/LBM (kcal/day/kg)	0.027 (0.025–0.028)	0.028 ± 0.002	0.577
REE/TBW (kcal/day/kg)	13.5 ± 2.3	15.5 ± 2.2	**0.043**

*Values are presented as mean ± SD or as median (IQR). Excess body weight, weight – ideal body weight (50th for age). BMI, body mass index; REE, resting energy expenditure; LBM, lean body mass; TBW, total body weight. Bold values indicate p < 0.05*.

### Correlation of Weight Loss With Body Composition and REE After Sleeve Gastrectomy

In an effort to seek the factors that could affect weight loss, we evaluated the correlation between total percent weight loss and body composition and REE parameters at baseline (before surgery) and after surgery. There was no correlation between percent total weight loss and body composition and REE parameters at baseline. However, percent total weight loss was positively associated with REE/TBW at 12 months and negatively with fat mass and % fat mass at 12 months. This is shown in Table 5.

**Table 5:** Correlation of % total weight loss and body composition and measured REE before and after surgery.

	**Pre-surgical**		**Post-surgical**	
	**r/Rho**	***P*-value**	**r/Rho**	***P*-value**
**PERCENT OF INITIAL WEIGHT LOST**				
% Fat Mass	0.003	0.992	−0.669	**0.024**
Fat mass (kg)	0.271	0.490	−0.642	**0.033**
% Lean Mass	−0.105	0.759	0.576	0.064
Lean Mass (kg)	0.233	0.419	0.032	0.926
REE	−0.035	0.914	0.028	0.931
REE /LBM	−0.264	0.433	0.436	0.178
REE /TBW	−0.152	0.637	0.625	**0.03**

*Linear Correlation- Spearman's test for non-parametric and Pearson for parametric distribution. BMI, body mass index; REE, resting energy expenditure; LBM, lean body mass; TBW, total body weight. Bold values indicate p < 0.05*.

### Correlation of Changes in Measured and Estimated REE Before and After Sleeve Gastrectomy

To evaluate if the routinely used equations can at least be used to track the changes in REE before and after surgery, we ran a correlation analysis on the changes in REE obtained with indirect calorimetry (mREE at 12 months—mREE at baseline = ΔmREE) with the changes in estimated REE obtained by each of the four equations (eREE at 12 months—eREE at baseline). We found that Δ mREE with Δ Mifflin eREE (*r* = 0.48; *p* = 0.06); Δ mREE with Δ Harris-Benedict eREE (*r* = 0.41; *p* = 0.11); Δ mREE with Δ WHO eREE (*r* = 0.52; *p* = 0.04) and Δ mREE with Δ Deru eREE (*r* = 0.43; *p* = 0.10).

## Discussion

### Resting Energy Expenditure Equations in Adolescents and Young Adults With Severe Obesity

This study questions the utility of using predictive equations clinically to estimate REE in adolescents and young adults with moderate to severe obesity because of the high degree of variability in these predictions with a meaningful clinical error. Even the Mifflin equation, which provided the most reliable estimates, only accurately predicted REE in approximately one quarter of the subjects in our cohort. All four equations tended to overestimate REE with a high degree of bias (20–40%) above the measured REE. The Bland-Altman plots, which quantify the agreement between the two quantitative measures, showed reasonable agreement for all the equations tested (16). While these agreements are acceptable at the population level, the degree of discrepancy at the individual level limits the clinical utility of using the equations in guiding weight management treatment. The average calorie deficit recommended to achieve a weight loss of 1–2 pounds/week is about 500 kcal/day and most of these equations overestimated the individual calorie goal by that amount.

Previous scientific literature has also found that equations are inaccurate in predicting REE for adolescents with obesity (4, 11, 15, 17). However, our results seem to have even lower accuracy when compared to other studies. Even when looking at the same equation, for example, Derumeaux, our study found an accuracy prediction in 14.1% of subjects, compared to Marra et al. (4), who found accurate predictions in 43% of females and 48% of males. Lazzer et al. (15) also tested the Derumeaux equation, finding an accurate prediction in 22% of subjects. The results of our study, and in the context of these other similar studies, suggest that predictive REE equations should be used with caution in this age and weight group.

Similarly, other research has reported inaccuracies between measured and predicted REE in adults with obesity (18). Marra et al. evaluated 1851 adults with obesity to find that overall prediction accuracy was low. Testing 15 different equations, an average of 55% of subjects were found to have an accurate predicted REE. When compared with our results, the accuracy of REE prediction in adolescents with obesity is far lower, reaching a mean 12.9% between the four equations.

There are different reasons for the lack of accuracy of these equations and these should be considered before applying them to patients. For example, in our study, the Harris Benedict equation accurately predicted REE in 6.3% of subjects, overestimating REE in the majority (91.7% of subjects). Harris and Benedict developed this equation using data from normal-weight adults aged 20–70 in 1918, and the equation was not designed to predict REE in a younger population with excess weight. Though the Harris Benedict equation takes height, weight and age into account, other factors that contribute to REE such as fat free mass (11) and race (19) are not considered. In comparison, the Derumeaux equation includes fat free mass as a variable, but it was developed using data from children with obesity in a French population and does not take age into account for calculations in subjects. The ethnic diversity of our subjects may also contribute to the inaccuracy of this equation in predicting REE. Since REE can be affected by many factors at an individual level namely, lean mass, sleep, macronutrient composition of the previous meal and season of the year, it is extremely challenging to develop a precise equation at an individual level. Inaccuracy between measured REE and calculated REE has been reported in other studies of adolescents with obesity (4, 15, 17), and given these results we suggest using caution while using these equations to predict REE in adolescents with obesity unless validated in the specific population being assessed.

### Energy Expenditure, Calorie Counting, and Weight Loss

In clinical practice, an assessment of energy requirements has been a key component to guiding dietary recommendations for weight loss. The Academy of Nutrition and Dietetics, Intervention of the treatment of overweight and obesity in adults (20) suggest that the Mifflin equation should be used to estimate metabolic rate in adults, followed by a dietary intervention to decrease consumption of energy. The National Institute of Health (NIH) suggests a deficit of 500–750 kcal/day is required for weight loss. It provides a website and body weight planner based on the research by Hall et al. (21), with targeted deficits to achieve desired weight loss based on REE predictions. Eat for Health (22) is an initiative by the Australian Government that contains a similar calorie calculator. The initiative, in line with the Australian Dietary Guidelines and health promotion, suggests “*any energy intake above the estimated requirement is likely to result in weight gain.”* Furthermore, many gym and fitness programs use predictive equations to guide their programs. Equinox, a worldwide luxury fitness company uses REE equations in their initial consultations to help individuals achieve their specified fitness goals. Popular phone application “My Fitness Pal”, was founded in 2005 and has over 19 million monthly users. It aims to helps its users achieve their weight goals by asking them to input height, weight, gender and amount of desired weight loss, using predictive equations to give a daily net calorie goal as well as a specific date that your weight goal should be achieved. Weight Watchers uses a very similar approach, allocating an individual a number of “points” to eat per day based on the Mifflin equation. Textbooks such as Handbook of Clinical Nutrition and Dietetics (23) include whole chapters on predictive equations and how to use them.

New forms of technology including hand held indirect calorimeters and arm bands are being tested as a cheaper and more accessible option to measure REE accurately for continued use in the clinical setting (24, 25). On a different note, recent research suggests that perhaps the quality of calories ingested and not the quantity should be the primary focus for weight loss (26). In a study by Gardner et al. adult subjects with overweight and obesity were placed on a healthy diet with either low fat or low carbohydrate content and received instructional sessions every 2 weeks on healthy eating over 12 months, educating patients to eat more vegetables and less processed foods without following a caloric target. Subjects in both groups lost an average of 5–6 kilograms at 12 months regardless of diet in the absence of any caloric targets (26).

In addition to the inaccuracy of REE calculations, the accuracy of food labels, which are often used to estimate the caloric content, needs to be considered. Furthermore, the amount of calories extracted from any one food may differ from person to person. Studies have shown that our individual microbiome can affect digestion and the energy required to break down different foods (27). Thus, there are many variables that may impact the accuracy of calorie counting, leading to very crude estimations even in the hands of an astute patient.

The mental health effects of recommending calorie counting in adolescents should also be considered. Adolescents with obesity are at increased risk of developing an eating disorder and are more likely to use maladaptive behaviors to control weight (e.g., use of laxatives or induced vomiting) when compared to normal weight peers (28, 29). Students who diet by severely restricting their energy intake are at much greater risk of developing an eating disorder compared to those who do not diet (30).

Thus, given these inaccuracies in estimation of REE, compounded by the fact that it is difficult to get an accurate estimate of caloric intake and doing so may not be beneficial to the overall well-being of adolescents with obesity, we should challenge the utility of this practice. Providers, government recommendations and the health and fitness industry should move away from inaccurate energy expenditure calculations, calorie goals, and weight targets, and instead focus on promoting healthy lifestyle habits.

### Changes in Energy Expenditure After Bariatric Surgery

Bariatric surgery is an effective weight loss tool (31), which results in long term weight loss and improvement in metabolic outcomes across the age spectrum (32). There are many factors that affect weight loss and weight regain after surgery, and alteration in REE is postulated to be one of these factors. Sleeve gastrectomy is currently the most commonly used bariatric procedure. To our knowledge, this is the first study that evaluates changes in REE after SG in adolscents (33). Our results show that absolute REE decreases after SG, consistent with reductions in total lean mass. When comparing these changes to a historical cohort where adults with obesity lost weight by lifestyle intervention (The Biggest Loser), we found a smaller reduction in REE in our cohort who underwent SG. After 7 months, Knuth et al. found that participants of the Biggest Loser lost 35 ± 7.1% body weight, associated with a 617 kcal/day reduction in REE (7). Interestingly, our cohort who underwent SG, lost a similar percentage of body weight to the biggest loser participants, however had a far lesser decrease in REE after 12 months. The smaller reduction in energy expenditure after SG or a “blunting” of adaptive metabolic change may be an additional mechanism to explain how SG is more effective as a weight loss strategy than conventional diet/exercise weight loss over time.

Despite a reduction in absolute REE, REE controlled for total body weight increases after SG in adolescents. This is likely a reflection of the relatively greater decrease in metabolically inactive fat mass compared to metabolically active lean mass. We also found a very strong correlation between percent total weight loss and the ratio of REE/TBW, suggesting that this may be a significant factor that affects the degree of weight loss in adolescents after SG as seen in adults after gastric bypass (9). Further monitoring is needed to determine whether this contributes to weight loss maintenance and if it can be an early predictor of response to surgery.

Moreover, when we evaluated the changes in REE as obtained by indirect calorimetry before and after surgery with the changes estimated using the equations before and after surgery in our small cohort, we did not find a strong correlation. This suggests that the predictive equations are not reliable to even monitor the trend after sleeve gastrectomy. This finding further emphasizes the guarded use of these equations in a surgical setting as well.

Limitations of this study include the small sample size. Due to limited numbers, we were unable to assess the accuracy of predictive REE equations in specific ethnic groups. We were unable to compare metabolic changes in bariatric surgery with adolescents with those who lost a comparable amount of weight with diet and exercise alone, given the practical difficulty of losing 20–30% of body weight by lifestyle measures.

## Conclusion

Treating obesity remains a challenge. Predictive REE equations in adolescents with moderate to severe obesity have a considerable margin of error and should be used with caution in the clinical setting. It is time to shift away from calorie counting based on predictive estimates, given that these estimations are imprecise and may also be detrimental to the mental health of adolescents. Instead, providers should focus on promoting a healthy lifestyle in the absence of caloric targets. SG, the most commonly used bariatric procedure in adolescents, is associated with increases in REE after controlling for total body weight, likely from the proportionally greater loss of metabolically inactive fat mass and maintenance of lean mass. These changes in REE also correlate with percent total weight loss 1-year post-surgery.

## Author Contributions

All authors have made substantial contribution to the paper. FR, LT, SM, AT, FS, and VS were responsible for acquisition of data and data analyses. FS, VS, and HL were responsible for interpreting the data. FR, LT, and VS were responsible for drafting the manuscript. VS was responsible for the conception and design of the study and critical review of the paper. AB, SM, FS, MB, MM, and VS were responsible for revising the article critically and adding important intellectual content. MB, MM, and VS were responsible for acquiring the funding that sponsored the study. All authors have read and approved the final version of the paper.

### Conflict of Interest Statement

The authors declare that the research was conducted in the absence of any commercial or financial relationships that could be construed as a potential conflict of interest.
